# The experiences of individuals with cervical spinal cord injury and their family during post-injury care in non-specialised and specialised units in UK

**DOI:** 10.1186/s12913-020-05659-8

**Published:** 2020-08-24

**Authors:** Jackie McRae, Christina Smith, Anton Emmanuel, Suzanne Beeke

**Affiliations:** 1 Faculty of Health, Social Care and Education, Kingston and St Georges University of London, London, UK; 2 Division of Psychology and Language Science, University College London, London, WC1N 1PF UK; 3 Division of Medicine, University College London, London, WC1E 6JF UK

**Keywords:** Spinal cord injuries, Cervical cord, Deglutition disorders, Communication, Hospitalisation

## Abstract

**Background:**

Individuals with acute cervical spinal cord injury require specialised interventions to ensure optimal clinical outcomes especially for respiratory, swallowing and communication impairments. This study explores the experiences of post-injury care for individuals with cervical spinal cord injury and their family members during admissions in specialised and non-specialised units in the United Kingdom.

**Methods:**

Semi-structured interviews were undertaken with individuals with a cervical spinal cord injury and their family member, focussing on the experience of care across units. Eight people with spinal cord injury levels from C2 to C6, were interviewed in their current care settings. Six participants had family members present to support them. Interviews were audio-recorded and transcribed with data inputted into NVivo for thematic analysis.

**Results:**

The study identified six themes from the participant interviews that highlighted different experiences of care in non-specialised and specialised settings. A number of these were related to challenges with the system, whilst others were about the personal journey of recovery. The themes were titled as: adjustment, transitions, “the golden opportunity”, “when you can’t eat”, communication, and “in the hands of the nurses and doctors”.

**Conclusions:**

Whilst participants reported being well cared for in non-specialised units, they felt that they did not receive specialist care and this delayed their rehabilitation. Participants were dependent on healthcare professionals for information and care and at times lost hope for recovery. Staff in non-specialised units require training and guidance to help provide support for those with dysphagia and communication difficulties, as well as reassurance to patients and families whilst they wait for transfer to specialised units.

## Background

Spinal rehabilitation services have been well established over the last 20 years in UK and other developed countries, with recognised care pathways from admission to major trauma centres to spinal injury units (SIUs) following surgical stabilisation [1, 2]. Studies suggest improved outcomes for SCI patients managed in units that provide specialist rehabilitation, especially for those with cervical spinal cord injuries (CSCI) [3–5]. Many countries now implement early or direct admissions to SIUs to maximise recovery, however a UK report identified reduced SIU bed capacity nationally, with bed limits delaying admissions for those with additional ventilatory requirements [6]. The delay to accessing rehabilitation is associated with reduced quality of life and opportunity for independence as well as an increased risk of complications and healthcare cost [7].

The demographics of those admitted with SCI in the developed world, has changed over the last 20 years with an increase in cervical level injuries and an older population [8, 9]. High cervical level injuries are associated with respiratory impairments and an increased risk of complications for swallowing and communication functions, due to the need for surgery, tracheostomy and ventilation [10–12]. The presence of dysphagia increases the risk of pneumonia and malnutrition, which is associated with greater morbidity and mortality [13–15]. Early and direct access to specialist SIU ensures prompt identification and clinical management, reducing complications and improving outcomes for CSCI patients [16–18]. Maharaj et al. [19] identified significant improvement in outcomes after introducing a non-refusal policy to ensure admission to a SIU within 24 h. With limited SIU capacity in UK, an evaluation of the variations in care across specialised and non-specialised units identified staff differences in the management of respiratory problems and dysphagia, that may account for the change in outcomes [20]. It is not clear what impact this has on the experiences of people with CSCI who require specialist rehabilitation.

A component of the experience of specialist rehabilitation is the contribution towards restoring self-agency following disruption to physical ability and ‘life biography’ [21]. This relies on interventions delivered by specialist clinicians [17], as well as establishing relationships with healthcare staff that provide a holistic approach to care [22]. Psychosocial and emotional adjustments are influenced by support received, especially from family members [23] although increased caregiver burden has been identified in those caring for people with tetraplegia during and after rehabilitation with secondary complications being key factors [24, 25]. Despite this recognition, few studies have explored the distress experienced by family members during the acute phase, which may have significant impact on recovery.

Studies in a number of countries have focused on successful community adaptation of individuals post-spinal cord injury and identified a dependence on information, guidance and rehabilitation provided to them during their hospital rehabilitation period [26–29]. There is little information from the UK on the post-injury experiences of patients and families in different care settings and the impact of the transition between them. The aim of this study was to explore the views and experiences of individuals with CSCI and their families in the context of post-injury care in non-specialised and specialised units, with a specific focus on the management of additional complications of swallowing and communication difficulties.

## Methods

### Setting and study design

The study aimed to recruit ten participants to achieve data saturation. Participants were interviewed on-site of their current care environment, either an inpatient or residential facility based in the south of England. One-to-one semi-structured interviews took place between March and October 2015 with individuals with CSCI and their carer or family member. Interviews were carried out by the first author (JM) who is a speech and language therapist with 13 years’ experience working in a specialised spinal unit. An interview topic guide was developed based on key issues identified in the literature on SCI patient experiences. Questions were topic-based with open questions and prompts for elaboration or further recall. These were reviewed and approved by a patient advisory group for suitability and appropriacy (see supplemental material). Interviews were audio-recorded using an Olympus DM-901 digital voice recorder for transcription by an external agency and checked against the recording by the first author (JM). Thematic analysis was employed to identify emerging themes from the interview data [30].

### Participant selection

Sampling was purposive, with recruitment by healthcare professionals based in five specialist spinal units across England recruited as Participant Identification Centres (PIC). Inclusion criteria for invited participants were that they were English-speaking inpatients or outpatients aged over 18 years, who had sustained an injury to the cervical spinal cord at least three 3 months earlier, been diagnosed with dysphagia and required non-oral nutrition following injury. Participants with a tracheostomy who were unable to vocalise were included and adjustments made to ensure recordings of their interview were possible. Participants with cognitive or language impairments, pre-existing dysphagia or limited tolerance of the interview process were excluded. Individuals who met the inclusion criteria were sent written details of the study by their link healthcare professional with an invitation for participants to contact the first author by email, phone or reply-paid form. Following contact, a mutually convenient time was arranged for a face-to-face meeting for consent and interview. During this meeting, the study process was detailed to gain informed consent. This was recorded with a signature either by the participant or a proxy, if the participant was unable to sign but could verbally consent. Eleven invitations to participate were distributed by healthcare professionals from the PICs and nine expressions of interest were received. Reasons for non-participation were not explored as the lead author did not have direct access to these individuals.

### Data collection

Nine participants with a CSCI were interviewed in their care location. Prior to interview, participants were asked to provide demographic information which included the date of injury, level of SCI, and dates of admission and discharge for each specialised and non-specialised units to which they had been admitted.

Demographic characteristics are detailed in Table 1. Six participants had sustained their injuries following a fall, two from a sports injury and one from a non-traumatic injury following spinal surgery to excise a tumour. Two participants were female. The mean age at injury was 54 years. The mean time since injury was 12.3 months (range 5–27 months) and average time before admission to SIU was 7 months (range 2–16 months). Five participants had been in two ICUs and two had been in three ICUs before admission to a specialised SIU. One participant had not been admitted to a SIU and continued with rehabilitation in a residential setting.

**Table 1:** Demographic details of interview participants and status at interview

Pseudonym	Injury level	Age group at injury (years)	Aetiology	Respiratory/ communication status	Time since injury (months)	No. of ICUs prior to SIU admission (months)	Carer present Y/N
Margaret	C5	60–69	Fall (H)	V, T, SV	7	2 (5)	N
Roger	C2	60–69	Fall (L)	V, T, no speech	22	2 (16)	Y
Arthur	C6	70–79	Fall (L)	Normal	27	3 (8)^b^	Y
Keith	C4	70–79	Fall (L)	T, SV	8	2 (6)	Y
Paula	C4	20–29	Sports injury (H)	Aphonia	6	3 (2)	N
Simon	C4	30–39	Spinal tumour	T, SV	18	3 (14)	Y
Ryan	C7, T3	20–29	Sports injury (H)	Dysarthria	6	2 (3)	Y
George	C2/Central cord syndrome	70–79	Fall (L)	T, SV	5	2 (2)	Y
Thomas^a^	Don’t know	70–79	Fall (L)	Normal	12	1 (2)	N

^a^Excluded from group analysis; ^b^not admitted to a SIU.

*H* High impact injury, *L* Low impact injury, *V* Ventilated, *T* Tracheostomy, *SV* Speaking valve, *SIU* Spinal injury unit

At the time of interview, two participants were ventilator-dependent, and a total of five participants had tracheostomy tubes in situ, of which four used one-way speaking valves. Two participants were unable to use their own voice (due to aphonia and dysarthria respectively). Six participants had carers or family members present during the interview who were able to support communication attempts for those who were unable to generate a voice. One participant (Thomas) had difficulty during the interview in recalling his experiences post-injury and was unable to respond to subsequent questions and did not have any family members present to support him. His data was excluded from the final analysis. Data from eight interview transcripts were reviewed using thematic analysis to generate themes.

### Data analysis

Thematic analysis was conducted using NVivo for Mac Version 10.2.2 (QSR International) to manage the data. The process was undertaken by the first author (JM) and commenced with open coding of data from the first three interview transcripts to generate a set of codes, guided by the issues extracted from the literature that underpinned the topic questions. Where possible ‘in-vivo’ codes were used, (using the participants’ own words). These codes were then reviewed, grouped into categories and organised as themes. Each subsequent interview transcript was then coded using the established codes and categories, or new codes were generated if needed. An iterative process continued through review of the codes and earlier transcripts against newly generated themes until no new codes were generated. Rigour was achieved through discussion and agreement of coding and themes at two meetings with a peer group of three qualitative researchers, using transcripts to support the coding reliability process.

## Results

Six main themes were generated following thematic analysis of the transcripts: ‘adjustment’, ‘transitions’, “the golden opportunity”, “when you can’t eat”, ‘communication’ and “in the hands of the nurses and doctors”. The last of these main themes had three sub-themes; “This is it … and you’ll have to accept it”, ‘staff contact’ and ‘personal kindness’. Each of the themes will be detailed in turn with supporting quotations from participants who were given a pseudonym to allow anonymised reporting.

### Theme 1: adjustment

Participants reported having to make a significant adjustment to their injury and physical limitations. The theme of adjustment encompassed survival in the acute stages, through to longer-term physical changes and psychological adjustment, with support from family, friends and external organisations.

For many of the participants, early memories post-injury were blurred due to the need for sedation during this period. Family members were able to support these early memories with their narratives about the time immediately following injury. Survival was a key goal in the early adjustment to a traumatic injury. Ryan’s parents feared the worst as they travelled to the hospital after his accident and waited while he was in surgery. Knowing that death was a possible outcome helped to come to terms with the alternative reality of living with a spinal cord injury:

*“When he got to hospital, everybody was ready. Apparently, I’ve heard since that he had pretty much the best team available and everybody was available. He was in surgery for a very long time. Nobody thought he was going to survive … They were taking it hour by hour.” (Ryan’s mother)*

Paula could not remember what happened after her injury but reported being told about the events of her accident by her friends and family. Her account shows she understood how serious and life-threatening it had been:

*“They told me that a policeman saw me, gave me mouth-to-mouth...The ambulance came, they resuscitated me. I think I was breathing until I got to the hospital, then I stopped … they gave me oxygen … I don’t remember waking up … I couldn’t move anything apart from my eyes … That’s how I was communicating, I think I would blink for yes, look up for no. That was for a few days … My family told me they said I wouldn’t survive.” (Paula)*

For some, the relief of surviving was overshadowed by difficulties adjusting to a huge life change, such as the need for ventilation, as Margaret explained:

*“I didn’t understand anything about ventilators, and I couldn’t really come to grips with all that at all. In the early stages I didn’t really ask questions, and then afterwards I did, and began to understand all about it and what sort of ventilation and all the rest of it, but I didn’t at the beginning … I realised I was lucky to be alive, and just stayed like that, keep still and nothing else awful might happen.” (Margaret)*

The early post-injury time emerged as an intense experience for participants and their family members, as they had to quickly come to terms with a life-threatening injury that would have life-changing consequences.

### Theme 2: transitions

The theme of transitions relates to the experience of changes to care settings, which sometimes occurred multiple times during participants’ hospital journey. The first location of care was a trauma centre with intensive care staff focusing on survival. Post-acute care was expected to take place in a rehabilitation or specialised spinal unit. The transition to different environments meant that participants and their families had to develop an understanding of new processes, systems and staff through observation and questioning. This caused distress especially in unfamiliar hospital environments:

*“[Hospital 1] was the most awful place on this earth. Dreadful. Everybody was in green scrubs, so you didn’t know who was who … There just seemed to be all these masses of people. They didn’t take any particular interest in you. You know, in green. Nobody could answer any questions.” (Keith)*

When transfer to a specialised unit was delayed due to limited bed availability, participants would instead be transferred to a non-specialised unit to wait until a SIU bed could be arranged. Participants and their families had no control over or understanding of the processes involved but were aware that their high-level need for respiratory support restricted access to a SIU:

*“It was okay in [Hospital 1] they were not equipped to deal with spinal injuries. The plan was to transfer me to [Hospital 2], so, six months went by with no progress. They then decided to transfer me to [Hospital 2] ITU. There was no procedure to get to a specialist spinal unit … they didn’t have a ventilated bed … the goalposts changed, with the spinal units being divided in deciding various criteria to do with breathing and capacity (Roger)*

After waiting for a bed at a specialised unit for many weeks, some participants were suddenly transferred at short notice without preparation. Despite anticipating the transfer, the process of transition was no less challenging for participants’ and family members:

*Coming to [the spinal unit], George was not prepared for it. You were never explained about your spinal injury … you weren’t prepared for the shock of actually seeing patients in wheelchairs or told that there is a possibility you might need a wheelchair either permanently or temporary, whatever the outcome would be. (George’s wife)*

Participants felt there was a lack of information provided to them by staff, which would have helped them to prepare for the transition into a new setting.

*Might have been nice to have had a team because then they could have explained to me the ethos a little bit more, to just explain how it all worked … then I wouldn’t have found the adjustment as difficult. I think that's something they could do here. Because they must realise the way it's done at the beginning is slightly strange. It's probably not like many other hospitals, and when you're ill anyway, that sort of thing can be very odd. (Margaret)*

Many participants and families expected staff levels of skills and knowledge to be similar across hospitals. When they became aware of variations in different units, this generated anxiety about the care being delivered. Some family members took charge by increasing their own knowledge of care needs required to ensure safety:

*I mean, the spinal side, they had no idea … it was a poor handover between [Hospital 1] and [Hospital 2]. They tried to change your ventilation as soon as you got there. I had a feeling that … the nurse who actually took the handover didn’t really … listen, because the first thing, when I went in, he was up quite high in bed … and I said, “Excuse me, can I ask what angle he’s at?” So she told me “we keep all our ventilated patients … ” I said, “Sorry, but George is not a ventilated patient, George is a spinal patient”. So she then was flicking through all the pages, and the bed went down. (George’s wife)*

In summary, participants reported experiencing several transitions from trauma units to non-specialised facilities, then to specialised spinal rehabilitation units with little or no information to prepare them. They attempted to increase their own knowledge to protect from further distress.

### Theme 3: “The golden opportunity”

Participants and their families described losing out on specialist rehabilitation that could have improved outcomes following SCI. Specialist input was needed to help the recovery of those with speech and swallowing difficulties. Simon had waited over a year and remained without speech whilst cared for in three different units. Together with his wife they were still hopeful that he would get into at a spinal unit to access specialist rehabilitation to help progress his communication skills:

*“He was always … second on the list...Absolutely had to go to a spinal unit, because he wasn’t talking and I just knew he could. I don't know why I knew he could. I just knew he could speak. I also knew he needed the chance. He needed the opportunity, and he needed to go to a specialist centre that would do that. He was promised the spinal unit, and I just thought, ‘You keep to them [promises]. You put us on a list.’ He needs to have specialist treatment from people who know what they’re talking about with spinal injury and I’m not giving up until he’s had that … there was a golden opportunity, absolute golden opportunity, and it appeared to be slipping away out of our hands. Again, it all comes down to the desire for Simon to be able to speak.” (Simon’s wife)*

After 4 weeks of rehabilitation in the spinal unit, both Simon and his wife reported dramatic improvements in his speech, confirming her belief in the value of specialist rehabilitation to regain these functions to help him make his own decisions:

*They poked and prodded and did all sorts of stuff to you in the first 24 hours. Now you’re speaking, aren’t you? You’re off the ventilator 12-14 hours a day, cuff down, speaking. You’re much more in-charge of your own destiny. [Simon’s wife]*

Ryan transferred to a spinal unit 3 months after his injury, having previously been in two non-specialised units. He quickly noticed the difference between settings in terms of staff knowledge and rehabilitation input, which made him realise this had been lacking previously:

*“Very more intense and busy (sic). The physios … are more knowledgeable, and they’ll timetable. Never had a timetable before. So even if it’s not just physio, I’m busy doing other things on my timetable, every single moment. They keep me very busy.” (Ryan)*

Arthur was the only participant who was not admitted to a specialised unit and instead was transferred directly to a residential home after 8 months in hospital. Both he and his wife describe the consequences of not having had specialist rehabilitation on his upper limb function:

*My hands. Well, they didn’t do nothing in [Hospital 3]. When I got here [residential unit], every day they were putting my splints on, but they didn’t do no good. (Arthur)*

His wife added:

*They were hot on chest care [at Hospital 3]. They were the best you could ask for. And his hands just went by the by. Because he couldn’t move his arms either, I think they were thinking, well, why bother with them? I mean, he knows he isn’t going to walk, but that does upset him that he can’t use his hands. (Arthur’s wife)*

In summary, participants recognised the value of specialist spinal rehabilitation input to achieving improved function. However, their experiences highlight their concerns that delayed access might have reduced their only chance of improvement and recovery.

### Theme 4: “when you can’t eat”

All the participants in the study were nil by mouth for a period of time after their injury due to a diagnosis of oropharyngeal dysphagia. Many had reported that they did not understand the reason for their swallowing problems and were distressed when told that they may never be able to eat again. They reported how staff used different methods to evaluate swallowing ability, and this caused confusion about how the prognosis was decided. Keith’s wife recalled him failing multiple swallow tests, adding to their frustration:

*“ … they had tried giving you the swallow test with that blue dye, hadn’t they, several times at [Hospital 1] and it had come through … going down into your lungs … They just did so many tests, and they said, “Well we’ll see if you are strong enough to swallow now and things,” and they’d say, “Oh no.” And each time we got our hopes up, didn’t we, and then they’d say, “Sorry, nil by mouth still,”.” (Keith’s wife)*

Arthur’s wife felt these tests were not refined enough to be the basis of life changing decisions, and challenged their findings:

*“The swallow nurse come round and they done the water test. Well, they had a bit of water and a bit of orange squash … but he’d got a chest infection at the time, so when he got it and he coughed … I mean, I still say we don’t know whether he was coughing because he was swallowing, or coughing because he … got a cough. But then they decided that was it, he was never going to eat again or drink again.” (Arthur’s wife)*

After being told they were nil by mouth, participants struggled to consider a life without the pleasures of food and drink. Many reported little or no rehabilitation for their swallowing in the early stages, making them feel hopeless, especially as the routine of mealtimes provided structure to their day:

*“There was a time when you just passed the days but when you can’t eat, it isn't the same. You know, although eating is not maybe the most important thing to … it's fairly important, even if it's only a sandwich at lunchtime … when that's taken away, it's quite difficult.” (Margaret)*

Family members felt equally devastated to consider that one of the last remaining normal pleasures in life was now not possible:

*“ … but when we asked the consultant, and I said, “But how long will this be before Keith can have anything to eat or drink, or will his swallow improve?” he said, “Not ever” … and that was my blackest day, because Keith loves his food, don’t you? It was really grim. He said, “The swallow is very difficult to come back if that’s where the injury is.” He said, “I don’t think it will.” Which was awful, I thought. You know, everything else had been taken from him. Now just the thought of food and drink has been taken as well, whatever is life going to be like?” (Keith’s wife)*

Despite the possibility of recovery being dismissed in a non-specialist setting, some participants had their swallowing ability re-assessed at a specialised unit, many months later, and were provided with specialist rehabilitation to achieve recovery. Simon remained nil by mouth for 14 months before swallowing therapy allowed him to start eating again, whilst Margaret spent 6 months being nil by mouth, wondering if the long wait had been necessary:

*“The swallow test I had here [at spinal unit] was lots of different foods and then a big X-ray screen, and so this was the first food I'd had in 6 months, and all these tiny morsels of biscuits and oranges and all sorts of things. And they told me immediately, it was absolutely fine, you know, and I got the feeling that the doctor who was there nearly said to me, “I don’t know why you've been off food for so long.” The way he asked me questions and things, I felt it was all rather a waste of time, but I can’t be sure of that … I do wonder a bit; I think 6 months was far too long. I think there maybe was a time when I needed to be off food, but not as long as that.” (Margaret)*

The process of swallow recovery was gradual, with small trials of one texture before adding other textures and increasing amounts that helped to build a sense of pleasure and normality, as Paula describes:

*“They [SLT at hospital 3] came more than three times a week. So first they gave me something like three scoops then soft food, porridge, mashed potato, stuff like that. But I soon moved onto normal food. I called my mum to buy me Nandos.” (Paula)*

In summary, swallowing problems were an unexpected and distressing side effect of CSCI for participants, as this was their last remaining pleasure linked to normal life. There was confusion about the prognosis when participants had no clear reasoning for the problem, and many expressed huge relief when swallowing problems recovered after receiving specialist therapy.

### Theme 5: communication

This theme included reported attempts to interact with people in the environment, as well as reflections on not being able to talk. Communication with family and staff was a challenge for all participants whilst they could not use their own voice due to the need for tracheostomy and ventilation. Many participants relied on mouthing to communicate, which they reported as effortful and often unsuccessful:

*“If you can’t make a noise, you’ve lost the battle before you start … People thought I was getting cross with them, but I wasn’t. I was getting cross with myself because I couldn’t get the message across.” (Keith)*

*“It was difficult before because I couldn’t make a sound at all. So I had to mouth for ages. Some people can understand better than others but before it was a nightmare”. (Paula)*

*“I remember not being able to talk. And it was frustrating because I tried to talk, but nothing would come out. I got very frustrated that you [mum] couldn’t understand me.” (Ryan)*

Participants reported little early support to facilitate communication attempts, which increased their frustration and families recognised that this made them withdraw from interactions:

*“He got really, really annoyed. If I couldn’t understand him after two or three goes, it was, “Forget it, forget it.” I think he tended to sleep a lot when people were there because he couldn’t communicate.” (Arthur’s wife)*

Accessing alternative means of communication, such as writing, pointing or keyboard, was challenging for participants due to limited upper limb function. All communication was reliant on mouthing or eye-pointing with success dependent on support and problem solving by the communication partner:

*“Just a simple letter board with the alphabet. My dad made us just a piece of cardboard with the letters on and I’d write it down for him.” (Arthur’s wife)*

Simon relied on alternative communication for over a year after being unsuccessful at achieving speech because of his ventilation needs, and the low technology aid he was provided with limited him to conveying short pieces of information:

*“We were given the E-tran board [low technology aid]. Fairly early on you were taught with whoever it was how to use it, and then she taught that to us, how to use it … That worked very well. Very short answers, and if we did go with anything longer we started learning we’ve got to write it down.” (Simon’s wife)*

As he developed good eye pointing, he was provided with a high technology eye-gaze communication aid which allowed greater communication options and opportunities for interaction:

*“We then moved on to the TOBI computer for the special effect, and you were brilliant at the computer but you did get tired with using just the eyes, and stuff.” (Simon’s wife)*

In summary, communication in the hospital environment was a challenge for participants and family members, especially in the early stages when situated in a non-specialised unit. Participants used mouthing and low technology aids with reliance on a communication partner to enable them to generate a message. Communication options improved once participants were able to have their tracheostomy removed or adjusted and use their own voice. This usually took place with staff in the specialised spinal unit, although three participants still experienced ongoing communication difficulties.

### Theme 6: “in the hands of the doctors and the nurses”

The final theme captures participants’ experiences of being dependent on healthcare staff for information, care and support during their hospital stays. For many this was their first experience of inpatient care, and as their admission lasted many months their experiences changed over time. Consequently, three sub-themes were identified from the data: “This is it … and you’ll have to accept it”, staff contact, and personal kindness.

#### “This is it … and you’ll have to accept it”

Coming to terms with a life-changing prognosis of permanent disability following CSCI was often recalled as a negative experience, with compassion from staff not guaranteed. Participants and family members vividly recalled specific events and the strong emotions these prompted. Arthur’s wife commented:

*“The doctor turned round and said, “Of course you are not going to walk again. If you were going to walk again we’d have known that after 6 weeks.” and he walked out. And I hate that doctor for the way he said it. I mean, Arthur was upset anyway. And I’d got it in my head he wouldn’t by then. But to be told like that.” (Arthur’s wife)*

Keith remembered when a doctor told him very directly there was no hope for recovery:

*“They said to me, “This is it” he said, “no eating, immobile or anything else. This is it.” He said, “And you’ll have to accept it”. (Keith)*

He further recalled the discomfort of being nil by mouth, with some staff providing relief for his dry mouth while others enforced a no-water policy, not trusting him to spit out any residue:

*“You [were] very fortunate if you had any mouth care … Well, I used to plead for some water, but they wouldn’t give it to me … I was saying, “Let me have a mouthful of water, I’ll swill it round my mouth and I’ll either spit it out or suction it out.” And some would agree to it, some of them wouldn’t. And one night, this was the crux point for me, they came in and I said, “Could I have some water, my mouth is dry?” The nurse who’d come on for the night shift said, “It says nil by mouth, and that’s what it’s going to be”.” (Keith)*

#### Staff contact

Participants relied on healthcare staff to deliver care and provide them with information on their progress. Some participants reported having complete trust in their teams and did not feel they could challenge or question their actions:

*“I just put my hands in the hands of the nurses and doctors … because when you are in a hospital you just basically rely on what they do … I knew nothing and was just in their hands and hoping that they knew what they were doing. Trusting them.” (George)*

Both positive and negative experiences were reported, especially linked to the lack of consistency in staffing, which affected continuity of decisions and clinical progress:

*“There was one doctor who was really good and explained in normal words. Whereas sometimes you know what doctors are like and they explain in doctor words, it was like … but then a nurse would come up and say, “Well what they meant is … ”.” (Arthur’s wife)*

*“Every so often somebody would appear who was obviously a consultant, who would say, “Next week you’ll do this, you’ll do that.” Next week came and went, nothing happened. Because they were on this rota system, and a different consultant would be in charge every week … So you only saw the consultant every fourth or fifth week, by which time I’d forgotten what they’d said to me in the first place.” (Keith)*

#### Personal kindness

Many participants recalled positive experiences with staff who showed kindness. This sub-theme highlights the relationships that evolved over time, creating a personal approach to care. Personal kindness had a lasting and positive impact on participants as they tried to navigate an alternative existence over many weeks and months. Keith’s wife felt she was acknowledged as having her own needs after spending every day in the hospital:

*They were super. It was such a contrast … because if the tea trolley came round, they would give me a cup of tea … one of the staff nurses said, “There’s always things left over. To save you going home and then cooking a meal, would you like to have what the patients are having?” So I said, “Well that would be lovely, but am I allowed to do this?” and she said, “Well it’s only going to be thrown away.” (Keith’s wife)*

Ryan and his mum experienced caring that went beyond his physical needs, creating an unexpected friendship:

*He made such good friends … they kept popping back and then they’d see him. In fact, one of the nurses took his trache out … She was on a course, I think, that day and it was due to start at 9 o’clock. She said, “No, that’s the first thing I want to do.” So she came in at 8 especially to do it. She took his trache out and then she said she went away and just called everybody and said, “A brilliant thing, I’ve got rid of the trache.” (Ryan’s mum)*

In summary, the behaviour of staff made a significant impact on participants’ experiences of prolonged post-acute care. Communication and contact from staff often set the tone for the overall hospital experience. Kindness was usually attributed to a specific individual and was unexpected. It was a contrast to the daily challenges faced and significantly enhanced the experience of care.

## Discussion

This qualitative study provides a unique insight into the post-injury hospital experiences of eight people with CSCI and their families across non-specialised and specialised units in the UK, that are relevant to planning patient care pathways. In this study participants reported extended delays to transfer to SIUs, ranging from two to 16 months after injury. This in turn necessitated prolonged stays in at least two different ICU settings with care from staff without specialist spinal skills. Reasons for multiple ICU transfers was not always made clear and may have been for clinical needs and location. This study underlines the added demands made on participants and families to adapt to new clinical environments and staff relationships whilst adjusting to the impact of a life changing injury.

The delay in transfer to SIU has been found to have a negative impact on clinical outcomes for those with SCI [31–33]. However, few studies have highlighted the personal cost for individuals with CSCI and their families when high levels of specialist care are required. Family involvement has been described as a key factor in preserving the ‘essence’ of the previous self, enabling recovery for those with life-threatening injuries [34]. This study highlighted the commitment of family members of those with CSCI and their role in ensuring consistent care whilst maintaining hope during their prolonged hospitalisation.

The experience of swallowing problems was distressing for participants and family members. Few studies have reported on the personal experiences of not eating or drinking [35] and this study highlights how important these functions were for the process of recovery, adjustment and quality of life [36]. Participants described how the decisions to remain nil by mouth were made by staff without full explanation or consultation, reflecting a paternalistic attitude towards SCI care that has been reported in other clinical scenarios [37, 38]. The attitude of healthcare professionals has been found to have a big impact on the experience of ICU and early rehabilitation process [22, 39, 40]. Most participants subsequently made a successful return to eating and drinking, despite their earlier poor prognoses. This suggests that staff may have lacked knowledge of the recovery trajectory for those with CSCI and were unable to provide the hope for recovery that was needed in the early stages [41, 42]. Specialist training and information should be made available to staff in non-specialised units to help them support individuals and their families.

All the participants had experience of being ventilated through a tracheostomy during their admission and five participants still required a tracheostomy at the time of interview. They reported how this had a detrimental effect on their ability to communicate to staff and family, supporting the concept that communication is an essential component of controlling one’s environment and resuming ‘life biography’ as part of the recovery process [21]. Although methods for facilitating communication for those with a tracheostomy exist, this requires specialist clinical expertise which may be lacking in non-specialised units [43]. Alternative methods of communication commonly used in acute settings include picture or letter communication charts or writing boards that demand upper limb function [44]. As the findings of the study show, these aids are inaccessible for those with upper limb paralysis following CSCI and result in greater dependency on family and staff, increasing distress and isolation [45]. When participants were able to resume communication, they could ask staff questions and contribute to decisions about their care [46] giving them greater control and involvement in their rehabilitation.

A unique quality of this study was the inclusion of participants with no audible voice at the time of interview. Often these participants are excluded from research involvement or their experiences are reported retrospectively when their voice returns [47]. Capturing the experience of participants with ongoing communication difficulties ensures inclusion of their views which may differ from those who have recovered. There were technical challenges as interviews were audio recorded, so responses were attained through lip reading then spoken back to the participant for verification during the interview. It was noted that those without audible speech gave shorter responses to questions which may have reduced the richness of data about their experiences. Similarly, the presence of family members may have inhibited the candour of responses. An alternative option would be to train a neutral but informed communication partner to act in a facilitative role although this may inhibit sharing of personal emotional experiences. A future study may interview individuals with CSCI and their family members separately to compare viewpoints on personal experiences.

A limitation of the study was the recruitment of participants with high level care needs, this was a challenge as it was not possible to contact individuals directly, but through proxy individuals, usually healthcare staff, family members and carers. The interview arrangements were made through proxies so it was difficult to clarify whether the reasons for lack of involvement were due to issues of the proxy or individual. Despite recruiting sites being situated across England, participants involved in the study were based in sites in the south of England. Another limitation was the possibility of recall bias for the two participants who were interviewed more than 20 months after their injury. A larger UK study is warranted to broaden the findings from the current study, whilst qualitative studies in other healthcare systems would help to compare experiences of post-injury care in different settings.

## Conclusions

This study has reported on the challenging experiences of post-injury care for individuals with CSCI and their families across non-specialised and specialised units in the UK. Adjusting to the consequences of the injury was balanced with a need for hope whilst waiting to access specialist rehabilitation. Many participants spent extended times being unable to speak, eat or drink and were dependent on staff for care and information. Establishing better relationships with staff helped to resolve many emotional challenges. Whilst earlier transfer to specialist units may not be achievable in the short term due to limited capacity, providing education and training to staff in non-specialised units may help to resolve the challenges of dysphagia and communication problems and help to support individuals with CSCI and their families to achieve better outcomes.

## Supplementary information

**Additional file 1.**

## Data Availability

Audio and transcription data are stored securely in the Research Innovation Centre at the Royal National Orthopaedic Hospital (rnoh.research@nhs.net).

## Abbreviations

U.K: United Kingdom
SCI: Spinal cord injury
CSCI: Cervical spinal cord injury
SIU: Spinal injury unit
ICU: Intensive care unit
PIC: Participant Identification Centres

## References

[1] Smith M (2002). Efficacy of specialist versus non-specialist management of spinal cord injury within the UK. Spinal Cord.

[2] Donovan W, Carter R, Bedbrook G, Young J, Griffiths E. Incidence of medical complications in spinal cord injury: patients in specialised, compared with non-specialised centres. Paraplegia. 1984;22. 10.1038/sc.1984.46. 10.1038/sc.1984.46 6493795

[3] Cheng CL, Plashkes T, Shen T, Fallah N, Humphreys S, O'Connell C (2017). Does specialized inpatient rehabilitation affect whether or not people with traumatic spinal cord injury return home?. J Neurotrauma.

[4] Maharaj MM, Hogan JA, Phan K, Mobbs RJ (2016). The role of specialist units to provide focused care and complication avoidance following traumatic spinal cord injury: a systematic review. Eur Spine J.

[5] Sumida M, Fujimoto M, Tokuhiro A, Tominaga T, Magara A, Uchida R (2001). Early rehabilitation effect for traumatic spinal cord injury. Arch Phys Med Rehabil.

[6] Spinal Injuries Association. A Paralysed System? https://www.spinal.co.uk/wp-content/uploads/2015/11/SIA-APP-Paralysed-System-Report-FINAL-lo-res.pdf 2015 (Accessed 18 July 2015).

[7] Merritt CH, Taylor MA, Yelton CJ, RS K (2019). Economic impact of traumatic spinal cord injuries in the United States. Neuroimmunol Neuroinflammation.

[8] Devivo MJ (2012). Epidemiology of traumatic spinal cord injury: trends and future implications. Spinal Cord.

[9] McCaughey EJ, Purcell M, McLean AN, Fraser MH, Bewick A, Borotkanics RJ (2016). Changing demographics of spinal cord injury over a 20-year period: a longitudinal population-based study in Scotland. Spinal Cord.

[10] MacBean N, Ward E, Murdoch B, Cahill L, Solley M, Geraghty T (2006). Characteristics of speech following cervical spinal cord injury. J Med Speech Lang Pathol.

[11] Chaw E, Shem K, Castillo K, Wong SL, Chang J (2012). Dysphagia and associated respiratory considerations in cervical spinal cord injury. Topics Spinal Cord Injury Rehab.

[12] Brady S, Miserendino R, Statkus D, Springer T, Hakel M, Stambolis V (2004). Predictors to dysphagia and recovery after cervical spinal cord injury during acute rehabilitation. J Appl Res.

[13] Martin ND, Marks JA, Donohue J, Giordano C, Cohen MJ, Weinstein MS (2011). The mortality inflection point for age and acute cervical spinal cord injury. J Trauma.

[14] Chamberlain JD, Meier S, Mader L, von Groote PM, Brinkhof MWG (2015). Mortality and longevity after a spinal cord injury: systematic review and meta-analysis. Neuroepidemiology..

[15] Berlly M, Shem K (2007). Respiratory management during the first five days after spinal cord injury. J Spinal Cord Med..

[16] New PW, Simmonds F, Stevermuer T (2011). Comparison of patients managed in specialised spinal rehabilitation units with those managed in non-specialised rehabilitation units. Spinal Cord.

[17] Parent S, Barchi S, LeBreton M, Casha S, Fehlings MG (2011). The impact of specialized centers of care for spinal cord injury on length of stay, complications, and mortality: a systematic review of the literature. J Neurotrauma.

[18] Middleton PM, Davies SR, Anand S, Reinten-Reynolds T, Marial O, Middleton JW (2012). The pre-hospital epidemiology and management of spinal cord injuries in New South Wales: 2004–2008. Injury..

[19] Maharaj MM, Stanford RE, Lee BB, Mobbs RJ, Marial O, Schiller M (2017). The effects of early or direct admission to a specialised spinal injury unit on outcomes after acute traumatic spinal cord injury. Spinal Cord.

[20] McRae J, Smith C, Beeke S, Emmanuel A. Oropharyngeal dysphagia management in cervical spinal cord injury patients: an exploratory survey of variations to care across specialised and non-specialised units. Spinal Cord Series and Cases. 2019;5(1). 10.1038/s41394-019-0175-y. 10.1038/s41394-019-0175-y PMC6474233 31240124

[21] Bourke JA, Hay-Smith EJ, Snell DL, DeJong G (2015). Attending to biographical disruption: the experience of rehabilitation following tetraplegia due to spinal cord injury. Disabil Rehabil.

[22] Garrino L, Curto N, Decorte R, Felisi N, Matta E, Gregorino S (2011). Towards personalized care for persons with spinal cord injury: a study on patients’ perceptions. J Spinal Cord Med.

[23] Post MWM, Van Leeuwen CMC (2012). Psychosocial issues in spinal cord injury: a review. Spinal Cord.

[24] Conti A, Clari M, Nolan M, Wallace E, Tommasini M, Mozzone S (2019). The relationship between psychological and physical secondary conditions and family caregiver burden in spinal cord injury: a correlational study. Top Spinal Cord Inj Rehabil.

[25] Backx APM, Spooren AIF, Bongers-Janssen HMH, Bouwsema H (2018). Quality of life, burden and satisfaction with care in caregivers of patients with a spinal cord injury during and after rehabilitation. Spinal Cord.

[26] Babamohamadi H, Negarandeh R, Dehghan-Nayeri N (2011). Barriers to and facilitators of coping with spinal cord injury for Iranian patients: a qualitative study. Nurs Health Sci.

[27] Fuseini A-G, Aniteye P, Alhassan A (2019). Beyond the diagnosis: lived experiences of persons with spinal cord injury in a selected town in Ghana. Neurol Res Int.

[28] Ide-Okochi A, Yamazaki Y, Tadaka E, Fujimura K, Kusunaga T (2013). Illness experience of adults with cervical spinal cord injury in Japan: a qualitative investigation. BMC Public Health.

[29] Nunnerley JL, Hay-Smith EJC, Dean SG (2013). Leaving a spinal unit and returning to the wider community: an interpretative phenomenological analysis. Disabil Rehabil.

[30] Braun V, Clarke V (2006). Using thematic analysis in psychology. Qual Res Psychol.

[31] Scivoletto G, Morganti B, Molinari M (2005). Early versus delayed inpatient spinal cord injury rehabilitation: an Italian study. Arch Phys Med Rehabil.

[32] Amin A, Bernard J, Nadarajah R, Davies N, Gow F, Tucker S (2005). Spinal injuries admitted to a specialist Centre over a 5-year period: a study to evaluate delayed admission. Spinal Cord.

[33] Middleton JM, Sharwood LN, Cameron P, Middleton PM, Harrison JE, Brown D (2014). Right care, right time, right place: improving outcomes for people with spinal cord injury through early access to intervention and improved access to specialised care: study protocol. BMC Health Serv Res.

[34] Ogilvie R, McCloughen A, Curtis K, Foster K (2012). The experience of surviving life-threatening injury: a qualitative synthesis. Int Nurs Rev.

[35] Segaran E (2006). Returning to normal: The role of eating in recovery from a critical illness. BJNN.

[36] Whalley HK (2007). Quality of life after spinal cord injury: a meta-synthesis of qualitative findings. Spinal Cord.

[37] Engkasan JP, Ng CJ, Low WY (2015). Who decides? A qualitative study on the decisional roles of patients, their caregivers and doctors on the method of bladder drainage after spinal cord injury. Spinal Cord.

[38] Pellatt GC (2004). Patient-professional partnership in spinal cord injury rehabilitation. Br J Nurs.

[39] Hofhuis JG, Spronk PE, van Stel HF, Schrijvers AJ, Rommes JH, Bakker J (2008). Experiences of critically ill patients in the ICU. Intensive Crit Care Nurs.

[40] Russell S (1999). An exploratory study of patients' perceptions, memories and experiences of an intensive care unit. J Adv Nurs.

[41] Lohne V, Severinsson E (2006). The power of hope: patients' experiences of hope a year after acute spinal cord injury. J Clin Nurs.

[42] Lucke KT (1999). Outcomes of nurse caring as perceived by individuals with spinal cord injury during rehabilitation. Rehab Nurs.

[43] MacBean N, Ward E, Murdoch B, Cahill L, Solley M, Geraghty T (2009). Optimizing speech production in the ventilator-assisted individual following cervical spinal cord injury: a preliminary investigation. Int J Lang Commun Disord.

[44] Patak L, Gawlinski A, Fung NI, Doering L, Berg J, Henneman EA (2006). Communication boards in critical care: patients' views. Appl Nurs Res.

[45] Radtke JV, Baumann BM, Garrett KL, Happ MB (2011). Listening to the voiceless patient: case reports in assisted communication in the intensive care unit. J Palliat Med.

[46] Happ MB, Garrett K, Thomas DD, Tate J, George E, Houze M (2011). Nurse-patient communication interactions in the intensive care unit. Am J Crit Care.

[47] Carroll SM (2007). Silent, slow Lifeworld: the communication experience of nonvocal ventilated patients. Qual Health Res.

