# Supplementary material for: The experiences of individuals with cervical spinal cord injury and their family during post-injury care in non-specialised and specialised units in UK
Source: BMC Health Serv Res. 2020 Aug 24;20:783. doi: 10.1186/s12913-020-05659-8 (PMC7443811; doi:10.1186/s12913-020-05659-8)
Supplement: Supplementary file 1 — Additional file 1. [file 12913_2020_5659_MOESM1_ESM.docx]

| **Topics** | **Questions** | ***Prompts*** |
| --- | --- | --- |
| **Patient history** | Could you tell me about the events leading up to your admission to the ICU?  Could you tell me about your stay on the ICU, as you remember it?  How long did you stay in ICU?  When did you get transferred?  Where to? | *how long you were there and what procedures you experienced?*  *If poor direct recall, what have you been told?*  *If don’t know, can I ask your family member about your time in ICU?* |
| **Ventilation and tracheostomy experience** | Can you tell me how things progressed with your breathing?  Did you need a breathing tube or help to breathe (from a ventilator)?  Can you tell me what that felt like?  Was there a ‘tracheostomy team’ that dealt with your tracheostomy?  Did you understand why you needed it?  Do you feel it caused you problems? | *Poor recall – request to ask family member about the situation at the time.*  *If tube still in situ, adapt questions to discuss difference with ongoing care.*  *Give time for patient to reflect on their experience and recall examples, which add richness to data.* |
| **Communication**  **ability** | Did you have difficulty being able to communicate whist in ICU?  Can you tell me how this made you feel?  How did you resolve these problems?  Were you offered any communication aids?  Were you seen by a Speech and Language Therapist? | *Valuable to explore whether patient found their own solutions or needed guidance.*  *Probe whether the physical restrictions were an added challenge that made access to communication charts difficult?* |
| **Feeding – oral intake vs. nasogastric/PEG tube** | Was there any time that you were told you weren’t allowed to eat or drink?  Did you need to be fed through a tube? Was that in your nose or into your stomach?  Was the process explained to you?  Were you seen by any specialists or therapists to investigate your swallowing?  How did you feel about that process and the decisions made? | *Involvement in decision-making?*  *Important to reflect on personal impact on the restriction of oral intake* |
| **Mouthcare** | Did you have your teeth cleaned regularly, when you wanted whilst you were in ICU?  Did you have any problems with either too much or too little saliva in ICU? How was that managed? | *Are they able to brush their own teeth? Are they satisfied with the way another person cleans?*  *Any preferred tools?* |
| **Things to Improve?** | Tell me is there anything that you feel could have been improved in your care?  Do you have any questions for me? | *Participants are free to reflect on their hospital admission and generate an idea from their own perspective that they consider would have a more positive impact.* |

Semi-structured interview topic guide
